# Loss of function of T-box 3 in the liver protects against MASLD

**DOI:** 10.1172/JCI197143

**Published:** 2025-09-16

**Authors:** Jacquelyn J. Maher

**Affiliations:** Department of Medicine and Liver Center, UCSF, San Francisco, California, USA.

## Abstract

The hallmark feature of metabolic dysfunction-associated steatotic liver disease (MASLD) is hepatic lipid accumulation. A recent search for genes impacting MASLD in mice uncovered the transcriptional repressor T-box 3 (*Tbx3*) as a top hit. In this issue of the *JCI*, Mannino et al. investigated the mechanism of action of TBX3 in murine MASLD. *Tbx3* deletion protected against MASLD by inducing high density lipoprotein binding protein and stimulating hepatic VLDL secretion. Loss-of-function mutations in human TBX3 identified in MASLD patients displayed a similar protective effect. Collectively, these findings highlight the importance of lipid export in the prevention of MASLD and identify a transcriptional pathway controlling hepatic lipid secretion that is poised for further investigation.

## Screening for adaptive gene variants in MASLD

Metabolic dysfunction-associated steatotic liver disease (MASLD) is the leading cause of liver disease in the world today, affecting one-third of the global population (1). MASLD poses a public health threat because of its potential to progress — often silently — to steatohepatitis (MASH), liver fibrosis, and hepatocellular carcinoma (2). Numerous research strategies have been employed to investigate the pathogenesis of MASLD, but a technique termed MOSAICS (method of somatic AAV-transposon in vivo clonal screening) recently developed by Hao Zhu and colleagues at UT Southwestern stands out for its creativity.

The principle behind MOSAICS is to introduce somatic mutations in mouse liver cells and then evaluate their contribution to liver disease by monitoring the ability of mutant cells to survive and clonally expand during experimental liver injury. MOSAICS utilizes an adeno-associated virus (AAV)-mediated gene delivery system that supports integration of the cargo of interest into the host genome and permits lineage tracing of the mutations over time. In previously published work, the Zhu laboratory used MOSAICS to investigate MASLD by delivering a library of single guide RNAs targeting genes pertinent to MASLD into the livers of Cas9-expressing mice. Placing these mice on a MASLD-promoting Western diet (WD) for 6 months led to the clonal expansion of cells whose loss-of-function variants protected against disease (3). The top hits from this study were all genes with known relevance to MASLD, which established the validity of the approach.

Armed with this information, Zhu and colleagues extended their studies to interrogate transcription factors and epigenetic proteins that may influence MASLD (3). In this extended work, MOSAICS identified the transcription factor T-box 3 (TBX3) as a regulator of hepatic steatosis, which was noteworthy as TBX3 had not been previously linked to MASLD. This finding prompted further study of the mechanism of action of TBX3 in metabolic liver disease, which is the subject of the work by Mannino et al. that is highlighted in the current issue of the *JCI*.

## TBX3 regulates hepatic lipid homeostasis in murine MASLD

As a prelude to studies of TBX3 in mice, Mannino and colleagues (4) first examined the livers of patients with MASLD to determine whether mutations in TBX3 are detectable in the human disease. They identified TBX3 mutations in four MASLD subjects, some of which localized to the DNA binding domain. Reviewing transcriptional data from a human database, they reported an increase in TBX3 expression in early MASLD followed by a decline as the disease progresses; they found a similar biphasic change in mouse liver in response to a 36-week WD. Seeing this similarity prompted the authors to proceed with studies of deletion or overexpression of *Tbx3* in mouse liver to determine its impact on the development of experimental MASLD.

**Figure 1:**
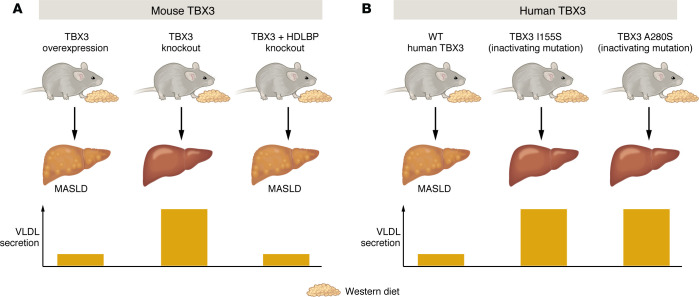
(**A**) Mice engineered to overexpress TBX3 in the liver developed MASLD in response to WD. By contrast, genetic deletion of TBX3 from the liver (TBX3 knockout) protected mice from diet-induced MASLD. The protection from MASLD seen in TBX3-knockout mice coincided with increased VLDL secretion. TBX3 knockout resulted in upregulation of HDLBP (not shown); when HDLBP was also deleted (TBX3 + HDLBP knockout), the protective effect of TBX3 knockout was eliminated coincident with lower VLDL secretion. (**B**) Two point mutations in TBX3 (I155S and A280S) that localize to the DNA binding domain and presumably suppress transcriptional activity were identified in MASLD patients. When these mutations were expressed in mouse liver followed by WD feeding, they behaved similarly to TBX3 knockout in mice, protecting against diet-induced MASLD and enhancing VLDL secretion.

Consistent with their earlier observations (3), the authors found that AAV-mediated deletion of *Tbx3* in the livers of mice reduced features of MASLD following WD feeding (Figure 1A). Most pronounced was a reduction in hepatic steatosis, with a lesser reduction in liver injury as measured by plasma alanine aminotransferase. Conversely, AAV-mediated overexpression of *Tbx3* in mouse liver followed by 3 months of WD prompted an increase in hepatic lipids as well as an increase in plasma transaminases. This exacerbation of diet-induced MASLD did not coincide with any change in insulin sensitivity.

Investigating how liver-specific *Tbx3* deletion could result in an improvement in hepatic lipid homeostasis during a WD challenge, the authors found that loss of *Tbx3* led to an induction of several genes involved in hepatic lipid secretion, and specifically genes regulating the synthesis of lipids and proteins involved in VLDL assembly. In accord with this observation, the authors showed that liver-specific *Tbx3* deletion stimulated hepatic VLDL secretion, resulting in elevations of plasma triglyceride and apolipoprotein B (ApoB) (Figure 1A). To verify that the enhancement of VLDL secretion in *Tbx3*-deficient mice was directly responsible for their protection from WD-induced MASLD, the mice were then fed a different MASLD-inducing diet deficient in methionine and choline (CDAHFD; choline deficient, amino acid supplemented high fat diet). The CDAHFD causes hepatic steatosis by limiting the chemical building blocks necessary for VLDL production (5, 6), and thus should not be salvageable by genetic means. Indeed, the beneficial effect of *Tbx3* deficiency seen with WD-induced MASLD was nearly completely abrogated in CDAHFD-induced MASLD. The observation that TBX3 regulates hepatic lipid secretion is noteworthy because perturbations in other genes integral to lipid export have also been implicated in MASLD. Noteworthy among these is a single nucleotide polymorphism in transmembrane-6 superfamily member 2 (*TM6SF2*), which is strongly associated with MASLD risk (7–10). Importantly, although the disease-promoting variant of TM6SF2 (E167K) suppresses hepatic triglyceride secretion, it does so without affecting ApoB secretion, meaning that affected patients still secrete VLDL particles but with low lipid content (11–13). TBX3 deficiency, by contrast, prevents MASLD by stimulating the secretion of both triglyceride and ApoB, suggesting that it increases the number of VLDL particles released into the circulation. Thus, TBX3 and TM6SF2 appear to regulate hepatic lipid secretion through independent mechanisms.

## Human TBX3 variants preserve lipid secretion in mice

The study team then explored which transcriptional targets of TBX3 might underlie the observed alteration in hepatic lipid secretion. Because TBX3 is predominantly a transcriptional repressor, they compared all genes bound by TBX3 to those upregulated in WD-fed *Tbx3*-knockout livers. A promising target from this analysis was high density lipoprotein binding protein (HDLBP), a candidate with known effects on secretory processes including VLDL secretion (14, 15). After verifying that *Tbx3* deletion induces *Hdlbp* expression*,* the authors demonstrated that deleting *Hdlbp* in a *Tbx3*-deficient liver was sufficient to eliminate the beneficial effect of *Tbx3* deletion on hepatic lipid secretion (Figure 1A). This validated the existence of a lipid secretion axis whereby a decrease in TBX3 function upregulates HDLBP, causing increased secretion of hepatic lipids.

In a final series of experiments, the authors returned to the TBX3 mutations they originally identified in human MASH livers to determine if they also influence lipid secretion. Two specific mutations in the DNA binding region (I155S and A280S) interfered with the nuclear localization of TBX3, and thus presumably inhibited its transcriptional activity. To test this hypothesis, the authors expressed the mutant proteins in H2.35 murine hepatocytes and demonstrated that they enabled secretion of a model compound better than wild-type TBX3. They then overexpressed the two TBX3 mutants in mouse liver in vivo, and demonstrated that triglyceride secretion was preserved after WD feeding, where it was suppressed in mice with wild-type TBX3. Moreover, mice with mutant TBX3 developed less hepatic lipid accumulation than those with wild-type TBX3, similar to the effect seen in *Tbx3*-deficient mice (Figure 1B).

## VLDL secretion emerges as a pathway of interest

This study underscores the key concept that dysregulation of hepatic lipid secretion is a critical feature of MASLD pathogenesis. To date, GWAS have identified numerous variants that pose a risk for MASLD; although these variants influence a number of metabolic processes such as intestinal lipid absorption, insulin sensitivity, hepatic lipogenesis and mitochondrial respiration (9, 10), the two variants with the highest significance in nearly all GWAS — *PNPLA3* (patatin-like phospholipase domain-containing protein 3) and *TM6SF2* — both affect hepatic lipid disposal (16, 17). Importantly, metabolomic studies also indicate that roughly 50% of persons with MASLD exhibit a phenotype characteristic of reduced VLDL secretion (18). Taken together, these observations identify the VLDL secretion pathway as worthy of special scrutiny for mechanistic discovery and therapeutic intervention in MASLD. The process of VLDL assembly and secretion is complex, and thus several control points are potentially implicated in MASLD pathogenesis. Indeed, variants in *TM6SF2* as well as microsomal triglyceride transfer protein (*MTTP*) and *APOB* have already been identified as MASLD risk factors having direct influence over VLDL secretion (7–10), and the current experiments add *Hdlbp* to this list. Unfortunately, from a translational perspective, the authors have already considered but dismissed the prospect that TBX3 suppression could be developed as a therapy for MASLD. The reason is that despite its beneficial effect on hepatic steatosis, *Tbx3* deletion promotes insulin resistance and hypertriglyceridemia in mice.

A second takeaway is that TBX3, unlike MTTP and TM6SF2, is a transcription factor, and thus has the potential to regulate more than one gene in the VLDL secretory pathway. The authors selected *Hdlbp* for further exploration in the current study, but did not elaborate on the existence of other relevant TBX3 targets. It is conceivable that *Hdlbp* is not the sole target of interest impacting hepatic lipid secretion.

Taken as a whole, the work of Mannino and colleagues stands out for its comprehensive analysis of TBX3, revealing a role for the transcription factor not only in MASLD through regulation of *Hdlbp* and VLDL but also in the general control of cellular secretion. In addition, the elegant reverse translation experiments with human TBX3 demonstrate its relevance to human liver disease. In the end, it is worth reflecting that TBX3 might not have become a target of interest in MASLD had it not been for its detection via MOSAICS as a gene whose loss of function ameliorates MASLD. The current study is an example of the combined power of MOSAICS and downstream analyses as drivers of discovery in liver disease.

## Version 1 09/16/2025

Electronic publication

## References

[1] Younossi ZM (2023). The global epidemiology of nonalcoholic fatty liver disease (NAFLD) and nonalcoholic steatohepatitis (NASH): a systematic review. Hepatology.

[2] Loomba R (2021). Mechanisms and disease consequences of nonalcoholic fatty liver disease. Cell.

[3] Wang Z (2023). Positive selection of somatically mutated clones identifies adaptive pathways in metabolic liver disease. Cell.

[4] Mannino G (2025). Somatic mutations in TBX3 promote hepatic clonal expansion by accelerating VLDL secretion. J Clin Invest.

[5] Yao ZM, Vance DE (1988). The active synthesis of phosphatidylcholine is required for very low density lipoprotein secretion from rat hepatocytes. J Biol Chem.

[6] Fast DG, Vance DE (1995). Nascent VLDL phospholipid composition is altered when phosphatidylcholine biosynthesis is inhibited: evidence for a novel mechanism that regulates VLDL secretion. Biochim Biophys Acta.

[7] Anstee QM (2020). Genome-wide association study of non-alcoholic fatty liver and steatohepatitis in a histologically characterised cohort. J Hepatol.

[8] Haas ME (2021). Machine learning enables new insights into genetic contributions to liver fat accumulation. Cell Genom.

[9] Vujkovic M (2022). A multiancestry genome-wide association study of unexplained chronic ALT elevation as a proxy for nonalcoholic fatty liver disease with histological and radiological validation. Nat Genet.

[10] Chen Y (2023). Genome-wide association meta-analysis identifies 17 loci associated with nonalcoholic fatty liver disease. Nat Genet.

[11] Smagris E (2016). Inactivation of Tm6sf2, a gene defective in fatty liver disease, impairs lipidation but not secretion of very low density lipoproteins. J Biol Chem.

[13] Liu J (2024). Basic and translational evidence supporting the role of TM6SF2 in VLDL metabolism. Curr Opin Lipidol.

[14] Mobin MB (2016). The RNA-binding protein vigilin regulates VLDL secretion through modulation of Apob mRNA translation. Nat Commun.

[15] Zinnall U (2022). HDLBP binds ER-targeted mRNAs by multivalent interactions to promote protein synthesis of transmembrane and secreted proteins. Nat Commun.

[16] Johnson SM (2024). PNPLA3 is a triglyceride lipase that mobilizes polyunsaturated fatty acids to facilitate hepatic secretion of large-sized very low-density lipoprotein. Nat Commun.

[17] Boren J (2020). Effects of TM6SF2 E167K on hepatic lipid and very low-density lipoprotein metabolism in humans. JCI Insight.

[18] Martinez-Arranz I (2022). Metabolic subtypes of patients with NAFLD exhibit distinctive cardiovascular risk profiles. Hepatology.

